# ‘Pregnancy Has Its Advantages’: The Voices of Street Connected Children and Youth in Eldoret, Kenya

**DOI:** 10.1371/journal.pone.0150814

**Published:** 2016-03-04

**Authors:** Juddy Wachira, Allan Kamanda, Lonnie Embleton, Violet Naanyu, David Ayuku, Paula Braitstein

**Affiliations:** 1 Academic Model Providing Access to Healthcare (AMPATH), Eldoret, Kenya; 2 College of Health Sciences, School of Medicine, Moi University, Eldoret, Kenya; 3 Moi Teaching and Referral Hospital, Eldoret, Kenya; 4 Dalla Lana School of Public Health, University of Toronto, Ontario, Canada; 5 Indiana University, Fairbanks School of Public Health, Department of Epidemiology, Indianapolis, Indiana, United States of America; 6 Regenstrief Institute, Inc., Indianapolis, Indiana, United States of America; University of Oxford, KENYA

## Abstract

**Objective:**

Little is known about the reproductive health or family planning needs of street-connected children and youth in resource-constrained countries. The study objective was to describe how street-connected children and youth (SCCY) in Eldoret, Kenya, perceive pregnancy.

**Methods:**

This qualitative study was conducted between August 2013 and February 2014. A total of 65 SCCY aged 11–24 years were purposively sampled from the three referral points: 1) A dedicated study clinic for vulnerable children and youth at Moi Teaching and Referral Hospital (MTRH); 2) Primary locations in which street children reside known as “bases/barracks”; and 3) Street youth community-based organizations. In-depth interviews and focus group discussions were audio recorded, transcribed, and translated into English. Content analysis was performed after thematic coding by 4 independent coders.

**Results:**

The majority of SCCY interviewed were male (69%) and sexually active (81.5%). None had gone beyond primary level of education. The strong desire for SCCY to go through conventional life experiences including marriage and child bearing was evident. Sub-themes around desired pregnancies included: sense of identity with other SCCY, sense of hope, male ego, lineage, source of income, and avoiding stigmatization. The desire for children was highly gendered with male SCCY more focused on their social status in the street community, while for females it was primarily for survival on the street. Female SCCY generally lacked agency around reproductive health issues and faced gender-based violence. Abortions (either assisted or self-induced), infanticide, and child abandonment were reported. Respondents described a lucrative market for babies born to SCCY and alleged that healthcare workers were known to abduct these babies following hospital deliveries.

**Conclusion:**

Our findings indicate gender differences in the reasons why SCCY become pregnant and have children. We also noted gender inequalities in reproductive health decisions. SCCY friendly interventions that provide tailored reproductive health services are needed.

## Introduction

In 2007, it was reported that between 250,000–300,000 children lived and worked on streets across Kenya.[1] Situational factors such as poverty, political instability, violence, physical/sexual abuse, being orphaned, drug abuse, unplanned pregnancies and family conflicts [2–6] predispose these children to street life. Once on the streets, street-connected children and youth (SCCY) are exposed to a hostile street environment that promotes early sexual debut, [7–10] physical abuse, and drug and alcohol use.[9,11,12]

There are a number of studies in Africa that have explored sexuality among street youth [7,10,13–17] and orphaned youth.[18–24] A recent study among SCCY in Eldoret Kenya, showed that initiation into the street community also contributes to engagement in risky behavior.[10] Additional studies among the same population revealed that unprotected sexual practices with multiple sexual partners which occurred as a result of rape, transactional sex, or desire for pleasure were common.[10,16] Furthermore, gender inequities in sexual practices exist, with street boys pursuing sex for pleasure, power, and dominance in the street social hierarchy while girls engage in sex predominantly as a survival mechanism.[10,16]

Outcomes such as unintended pregnancies [21] and sexually transmitted diseases including HIV are therefore common among this vulnerable group.[17,19,21,22,24,25] Unfortunately, Kenya like many other countries lack specialized health services for this population. We found limited studies that have explored pregnancy from the perspectives of SCCY. However globally, pregnancies among adolescents continue to pose a major public health concern. It is estimated that about 16 million adolescents (15–19 years) give birth each year, accounting for 11% of all births worldwide.[26] The majority (<50%) of these pregnancies occur in sub-Saharan Africa.[26] Given that these pregnancies are commonly unplanned and transpire among unmarried adolescent mothers, an estimated 2.0–4.4 million adolescents in this region undergo unsafe abortion each year.[26],[27] In Kenya, the national Demographic and Health Survey 2008–2009, indicated that the rate of teenage pregnancy in the country was 18%.[28] Efforts to reduce this rate even further are warranted.[28]

Generally, the factors associated with adolescent pregnancies in Kenya have been identified as being older in age, lower education level, peer pressure, lack of sex education, poor parental guidance, being orphaned, poverty and social environment-related factors including early marriages and sexual exploitation.[19,21,29] Early childbearing has negative health, social, psychological, and economic implications. Adolescence, the period between childhood and adulthood, accompanies profound physical and psychological changes that are not well developed for motherhood.[30] In addition, adolescents are less likely to be prepared for the social and economic responsibilities that come along with child bearing. The majority drop out of school due to their pregnancy,[28,31] which eventually limits their opportunity for financial stability and access to reproductive health services. Hence, maternal mortality and morbidity rates among adolescents are much higher compared to women over 20 years.[26,30] High morbidity, including low birth weight babies, and mortality rates are also reported among children born of adolescent mothers.[26,30]

Pregnant SCCY are faced with especially challenging circumstances given their poor health status[9,32] and environmental surroundings[5,10,33,34]. If we hope to develop evidence-based approaches to prevent early pregnancies, increase the use of contraceptives, prevent unsafe abortion, and increase the use of pre and post-natal care[26] among this group, a better understanding of how SCCY perceive pregnancy is fundamental. The objective of this study was therefore to describe how SCCY living in Eldoret, in western Kenya, perceive pregnancy.

## Methods

### Study Setting

Uasin Gishu (UG) County is one of the 47 counties in western Kenya. In 2010, UG County had approximately 894,179 individuals, of whom 41.5% were aged 14 years or less [35]. Approximately, 51.3% of the County population live below the Kenyan poverty line.[28] The headquarters of this County is Eldoret town, which has a population of 289,389.[28]

### Study Population

Our study population included ''full-time” and "part-time" SCCY aged 11–24 years who had lived on the street for more than 3 months. We considered "full-time" SCCY as those who lived on the streets on a full time basis or shared a shelter at night with other SCCY. While "part-time" SCCY were those who lived on the streets during the day and at night went home to an adult parent(s), relative or guardian.

### Human Subjects Protection

Ethical approval was obtained from the Moi Teaching and Referral Hospital (MTRH) Institutional Research and Ethics Committee (IREC) as well as the Indiana University Institutional Review Board (IRB). All interview sessions were conducted in private rooms. Prior to the interviews, trained research assistants provided SCCY with verbal information about the study and assessed their willingness to participate. Written consent was obtained from willing participants aged 18 years and older. Written assent was required for those aged 11–17 years, in addition to written guardian consent from the UG District children’s office. Fingerprints were used for children who were unable to sign or write their names. A child psychologist was present during all interviews sessions for children aged 11–17 years, in order to provide counseling in the event of psychological distress during the sessions. Privacy and confidentiality were assured at all times. Participants were requested to talk about their general perceptions and observations about sexual activities in the street community and not about their own personal experiences. In addition, participants were asked not to disclose their full names and/or those of other SCCY. First names of participants were used to facilitate discussions during focused group discussions (FGDs) sessions.

### Study design

This qualitative study employed in-depth interviews and FGDs with SCCY, and was conducted between August 2013 and February 2014. The objective of the study was to describe how the SCCY in Eldoret perceive pregnancy as groundwork for developing tailored appropriate interventions.

### Sampling and Recruitment

#### FGDs

FGD study participants were conveniently sampled from three points: 1) A dedicated study clinic for vulnerable children and youth at MTRH; 2) The primary locations that SCCY reside (bases/barracks); and 3) Street youth community-based organizations. Extensive street outreach and study sensitization occurred at these sites to establish rapport and trust with SCCY. A pre-existing relationship between the research team and a number of SCCY in Eldoret assisted in identification and outreach within these locations. A street outreach worker with experience working with this population was engaged to conduct the outreach and provide information about the study. Once the purpose of the study was explained, SCCY were invited to participate voluntarily in the investigation. Those who voluntarily came to the interview venue, which was the dedicated clinic for vulnerable children and youth, were recruited into the study after providing consent. To enhance homogeneity within the FGD groups, participants were stratified by age (11–13yrs, 14–17yrs and 18–24yrs) and sex (male vs. female). The different FGD sessions were held on the days convenient for the participants.

#### In-depth interviews

Participants for the in-depth interviews were purposively sampled from the FGD sample during the FGD sessions.

### Study instrument

A series of open-ended questions previously pre-tested among SCCY in UG guided the individual interviews and FGDs. The two main interview domains for this study included: 1) Sexual practices; and 2) Perceptions about reproductive health including contraceptive use and pregnancy. In addition, a set of structured questions on basic socio-demographic information including age, gender, educational level and area of residence were incorporated. An additional domain was included for interviews with barracks leaders that explored their role in the street community. The guides for the younger participants (11–13 years) were modified in order to reflect developmentally appropriate questions regarding sexual activities.

### Procedure

#### FGDs

Eligible participants were referred to the study clinic at MTRH by the street outreach worker where all interview sessions were conducted in private rooms. This location was chosen to provide a neutral, safe, and private environment where SCCY could feel more comfortable discussing their sexual and reproductive health practices. Once written consent or assent was obtained, participants were invited to take part in the FGDs which took an average of one and half hours. All sessions were audio-recorded and conducted by two of the study investigators (male and female) in Swahili, the Kenyan national language. These investigators were young (in their mid-30s) Kenyans with graduate level training in public health. They had a well-established rapport with the street community based on their involvement in previous health-related projects in Eldoret with this population. A trained research assistant was present in all sessions to take notes. At the end of the FGD sessions, the study team invited willing participants to engage in the in-depth interviews; scheduled on a later date that was convenient for the SCCY. In addition, transport reimbursement of Kenya Shillings 100 (~USD = 1.15) was provided. Given the cost that participants incurred in getting to the study clinic, this amount was considered adequate and not enough to coerce or influence participation in the study.

#### In-depth interviews

In-depth interviews took place in the same location as the FGDs and were conducted on a different day (a day convenient for the participants). Participants re-consented for the in-depth interviews which took on average 40 minutes. The sessions were audio recorded and facilitated by the same two investigators who conducted the FGDs sessions (one conducted 13 and the other 12 in-depth interviews). On completion of the interviews, participants were provided with the same amount of transport reimbursement as the FGD participants.

### Data Analyses

Recorded interviews were transcribed and translated to English. The data were then coded and themes related to pregnancy were identified. Concepts from different FGDs and interviews were then pooled together and integrated into common themes. Emerging themes were similar across both FGDs and in-depth interviews. Resultant themes were then used to organize the presentation of the results. For validation, independent coding and identification of themes were conducted by four investigators on four transcripts (two in-depth interviews and two FGDs). Once consistency was achieved in the coding, the four investigators were assigned equal number of transcripts to code independently. The final write up consisted of summaries, interpretations, and textual excerpts.

## Results

We conducted a total of 25 in-depth interviews and 5 FGDs (8–12 participants) with SCCY. Table 1 shows the distribution by session. Overall, we recruited 69 SCCY and included 65. One 14 year old boy declined to participate in the study while two participants (one female and one male, both 18 years old), could not participate in the study because they were intoxicated. The median age of those who participated was 18 years (IQR 14–20.5 years), with the majority (69.2%) being male. None had gone beyond primary education and only 24% were part-time SCCY. The majority (81.5%) reported themselves to be sexually active.

**Table 1:** Distribution of sessions.

Category	In-depth Interview sessions	FGDs sessions
Barrack leaders	4	0
Male		
11–13yrs	1	1
14–17yrs	6	1
18–24yrs	7	1
Female		
11–13yrs	0	0
14–17yrs	53	1
18–24yrs	4	1
** Total**	**25**	**5**

Generally, sexual relationships are established for monetary benefits, to fulfill sexual desire, or for marriage. Marriage among SCCY is an informal arrangement that involves a male SCCY identifying a house and requesting his sexual partner to move in with him. Word goes round about this arrangement suggesting that the marriage is formalized. However, ‘extramarital affairs’ among SCCY couples are common. To avoid their wives from engaging in extramarital affairs, male SCCY reported that they limit their wives’ interactions with the street community. On the contrary, female SCCY indicated they were independent during the day and returned home to their ‘husbands’ at night, suggesting that they still had contact with the street community.

Male In-depth Interview, Barracks Leader, 22 years: *When you get married at the base there are no weddings*. *You just get married*. *But when the man has money*, *maybe from selling stolen scrap metal*, *he will go and buy “busaa” (local brew) for everybody to drink and even those who want to smoke bhang (marijuana) will get it*. *That is how they get married*. *The difference will be*, *the husband will come to town to look for money and the wife remains home*.

Female In-depth Interviews, 21 years: *Yes there are marriages*. *They can get married then find a place that they will stay but they just go there at night to sleep and they spend the day at the base*. *Everybody is independent during the day but at night they go back together where the husband has rented a house*.

The main emerging themes around SCCY perceptions’ about pregnancy were: 1. Wanted pregnancies; 2. Unwanted pregnancies; 3. Unwanted infants; and 4. Preventing pregnancies. Under each theme, we established a number of sub-themes.

### Wanted Pregnancies

Pregnancy is highly encouraged; hence SCCY reported more advantages to pregnancy/child bearing than disadvantages. We noted some gender differences in the desire for SCCY to have children. SCCY male were more concerned about their social standing in the community while females bore children to please their partners, for economic purposes, and to avoid stigmatization. Shared sub-themes across both genders included sense of identity with other SCCY, sense of hope, and lineage.

#### Sense of identity with other SCCY

Having children on the streets brings about a sense of identity with other SCCY who have children. Since a considerable number of SCCY have either impregnated a girl, been pregnant, or have had offspring of their own, pregnancy or child bearing is considered normal. In addition, children are viewed as a symbol of love between a SCCY boy and girl that solidifies a sexual relationship. It also signifies that they are sexually active and are highly reproductive. SCCY often used the word ‘love’ when highlighting the desire for someone to want to have children with them. Therefore the stability of sexual relationships is heavily dependent on SCCY couples having children of their own.

Male In-depth Interview, 18 years: *If a girl loves a boy so much*, *she will tell him to give her a baby or she will go to someone who will*.

Female FGD, 14–17 years: *Yes it is good because it will make one to be called a mother*.

#### Sense of hope

Children are also seen to instill a sense of hope for a better future. SCCY believe that their children will end up living better lives than them, hence changing the course of their future. The presence of orphanages in the larger community assures the SCCY that their children will be educated and cared for.

Male FGD, 18–24 years: *They (referring to SCCY offspring) can be the future pastor*, *president*, *doctor*, *teachers…*. *so there is an advantage*.

Male In-depth Interview, 13 years: *There are some who are happy when they get pregnant*. *This means that they can get someone to help them*. *They are happy because they are assisted in bringing up the child till they are mature*. *The child is also educated*.

#### Lineage

Interestingly, SCCY were aware of the high mortality rates in their community. Having children therefore means that their lives are worth something and when they die they are assured that other SCCY will remember them through their children. Having children was also seen as a means of sustaining the number of SCCY living on the street.

Female FGD, 14–17 years: W*hen one dies*, *at least he/she will have a child to leave behind*. *For example if I die now*, *I don’t have even a child to leave behind*.

Male FGD, 18–24 years: *What makes me say I will have a child is because it is not good to die without leaving someone behind*. *Who will say I had a father called so and so*? *You leave behind a legacy with your children*. *I always say when I get a child*, *when I am blessed with a child*, *they won’t pass through the troubles I have passed through*. *Maybe that child is my savior*, *it was born to come and help me*. *Or if they go to school they will say*, *I once had a father*. *That makes many SCCY desire to get a child*. *So that they won’t die without their own legacy*.

Male FGD, 18–24 years: *Like me the way I am (SCCY)*, *when I get a woman from the streets*, *a person like me*, *a “mshefa” (Swahili word for hustler)*, *I always gamble with life*. *When I make that girl pregnant and she gets my baby*, *I can come to someone like the SCCY outreach worker and tell him to take care of the child*. *They should at least find a way to help my child go to school and become independent*. *Because I am a thief*, *I steal from people*. *I can be caught by the police or even killed by people*. *That child will remain with my name*. *People will say that is the child of so and so who was burned because of stealing*. *That is the child of a certain SCCY and he is in school*.

Male In-depth Interview, 20 years: *We get happy she is adding another member to our community*.

#### Male ego

Male SCCY experience a sense of pride when their sexual partners conceive or bear them children. They therefore expect their wives or girlfriends to have children with them in order to satisfy their ego. In addition, some SCCY males had a strong preference for a boy child compared to a girl child. Hence, female SCCY who are unable to conceive and more specifically a boy child, experience verbal and physical abuse from their male partners.

Male In-depth Interview, 20 years: *If I have a wife and she gets pregnant*, *I will be very happy because I want a baby…… and now I have a baby*.

Female In-depth Interview, Barracks Leader, 20 years: *For example if you are married and your husband sees other girls on the streets with children…*. *because most of the street girls give birth to boys*, *you will find your husband beating you up for no reason*. *He will later tell you that you don’t give birth*. *He will say “You don’t want to give me a child and other people’s (SCCY males) wives are giving birth*.*” So you just have to conceive and give birth so that you make him happy and he will not beat you*.

#### Source of income

Some SCCY use their children as a source of livelihood. Their experience is that strangers are more likely to give them money when they are carrying children. Hence, female SCCY sometimes give out their children to their peers, who pose as streets beggars. The money obtained from this activity is then shared amongst themselves.

Female, In-depth Interview, 21 years: *To add on the issue of children*, *most girls on the streets want to have children because it is a source of income*. *You will meet these women on the streets with children borrowing money*. *What they do is that if another person (SCCY female) doesn’t have a child*, *she will borrow a child then go with the child to the streets to borrow money*. *At the end of the day the money she gets will be shared with the mother when she returns the child to the mother*. *It is a very common business that is still going on to date*.

#### Avoid stigmatization

Given the value placed on having children, female SCCY who do not have children are stigmatized by their peers and referred to as ‘Tasa’ (Swahili word for barren). Pregnancy also signifies that female SCCY are eligible for marriage, hence female SCCY seeking male SCCY partners have to proof that they can give birth.

Female In-depth Interview, 21 years: I*f you don’t have a child on the streets*, *you will be verbally abused*. *They will abuse you and call you names like “barren” and other bad name*.

Male In-depth Interview, Barracks Leaders 18 years: *Yes there are such girls who don’t get pregnant*. *They are usually thought to be barren because they don’t get pregnant*… *people talk about her and state that she cannot get married*.

Very few disadvantages associated with pregnancy were cited. The disadvantages were linked to negative aspects of sexual intercourse, the delivery process, and caring for a child on the streets. Painful sex and sexually transmitted diseases, mainly HIV, were cited as disadvantages associated with sexual intercourse. Female SCCY mentioned that during delivery they experienced a lot of pain and there was a high possibility of them losing the baby in the process. Once with child, it was reported that some of the sexual partners of female SCCY did not want to accept the responsibility of taking care of the newborn. Hence, female SCCY were left with the entire burden of raising children on the streets by themselves. These sexual partners were either known (male SCCY) or unknown (random transactional sexual encounters) to them.

Female In-depth Interview, 15 years: *The problem is when you are impregnated by a person you don’t know very well*. *You don’t know where he comes from*, *his names and what he does*. *Sometimes you can even have sex with him in the lodging but you don’t know where he comes from*. *This makes it very difficult to trace him*.

Male In-depth Interview, 13 years: *A boy can make you (referring to a female) pregnant and deny having done it*. *You end up suffering not knowing where to go for help*.

Furthermore, female SCCY, many of whom are addicted to drugs, have a hard time meeting the basic needs of their children. It was reported that there were instances when infants were forcefully taken away from SCCY by hospital staff who believed that they were incapable of taking care of their babies. It was also reported that there were times where infants of SCCY were stolen from hospital maternity wards. To address this, a group of SCCY accompany expectant female SCCY during hospital deliveries and stay with them until discharge.

Male FGD, 18–24 years: *You know when a girl gets pregnant*, *life is hard and that is when you find many of the girls having their kids being taken away from them*. *You are a person who is dependent on alcohol*, *so you don’t know how to raise a child*. *You raise a child in poverty*. *So you find doctors taking children away from them (SCCY mothers)*. *Even when the children grow up*, *the majority of their mothers have died so the children even don’t know their mothers*.

Male FGD, 18–24 years: *To say the truth I had married one who came with an early pregnancy*. *We stayed together for one month*, *and she gave birth at the district hospital*. *The child was premature …I left my wife with the baby and went to town to look for money for the baby*. *My wife followed me to town but was caught and jailed*. *She was later released and I told her to come with her child and I would take of them…*.*she then told me that the baby was taken away from her at the maternity ward*.

### Unwanted pregnancies

Although SCCY cited a number of advantages associated with pregnancies, there were some pregnancies that were unplanned or undesired. Unwanted pregnancies are either terminated before term or dealt with once the infant is born. The majority of unwanted pregnancies are those that occur out of transactional sexual encounters, rape, or when a male SCCY abandons an expectant girl. In other cases, female SCCY are forced to terminate their pregnancy in order to establish new sexual relationships with male SCCY.

Female FGD, 18–24 years: *You can be influenced to perform an abortion by cunning men*. *You know a man can cheat and influence you to perform an abortion if it is not his baby*. *He can tell a pregnant girl to terminate the pregnancy because it is not his and so that they can to live together*. *That’s how you find a girl performing an abortion*… *Others also do that because the pregnancy is an outcome of rape*. *The girls say because it is out of rape*, *they can’t keep it since it will bring shame*. *So they abort*. *It can also happen when a couple fights… when the husband gets angry and chases his wife away*. *She can then decides to remove because it is her husband who made her pregnant*.

Abortion is either self-induced or executed by different people including healthcare workers, medical “quacks”, and traditional medicine men.

#### Health care workers

It was not clear who among healthcare workers actually performs this act but those SCCY interviewed mentioned instances when female SCCY would go to health facilities to have an abortion.

Female In-depth Interview, 17 years: *I usually see these hospitals* (referring to a number of hospitals) *perform abortions… I don’t know where it is specifically done because I have never done it myself*. *I have heard of so many girls who have come to hospital and performed an abortion*, *successfully*.

#### Medical quacks

Female SCCY have to sometimes part with as much as Kenya Shillings (Ksh) 5,000 (≅ 56 USD) to get an abortion from ‘medical quacks’. These individuals give the girls unspecified drugs including herbal medicine in order to terminate their pregnancy. Usually, they host the girls for a period of one week as they monitor the outcome of the abortion they have just performed. SCCY believe that abortion is safer when performed during the early stages of pregnancy.

Female FGD, 14–17 years: *The person who gives us herbs will stay with you to see if you will die because the process is risky*. *And you can part with as much as Ksh 5*,*000*. *You will not die if the pregnancy is still in the early stages*…*You stay with her for a week and usually it is a nurse who does it*.

#### Traditional medicine men

Medicine men also referred to as witchdoctors are sometimes consulted by the SCCY girls to perform the abortion.

#### Self-induced abortion

On a number of occasions, female SCCY induce abortion by ingesting drugs or concentrated fluids such as tea, juice, and detergent. Others adopt measures such as pressing on their stomachs, allowing someone to kick them in the stomach or pricking the fetus with a needle.

Female In-depth Interview, Barracks Leader, 20 years: *They just drink highly concentrated juice (quencher)*. *They put it in the cup then shake it vigorously to create a foam and then they drink it*. *Some will drink concentrated tea*. *You just put some little water in the sufuria and then add a lot of tea leaves*. *Then boil it as strong tea then drink it and that can help you abort*. *Some even drink omo (type of powdery detergent)*

Male FGD, 14–17 years: *The man kicks them (pregnant SCCY girl) in the stomach… When he kicks her*, *the girl miscarriages*.

Female In-depth Interview, 15 years: *Some girls will let the pregnancy to grow then when it reaches around 7 months*, *the fetus moves down and they start pricking the fetus with a needle through their vagina and labor pains begin then they end up having a still birth*.

Male In-depth Interview, Barracks Leaders, 18 years: *I have seen so many girls doing that*. *They just take some drugs that I don’t know where they get*. *I have seen so many aborted fetuses in town thrown in paper bags*. *I usually see these things when am just picking things*. *You pick a paper bag tied well and when you try to open it you find that the fetus is wrapped in*.

#### Multiple sexual partners

Interestingly, some of the SCCY believe that an abortion could occur if a pregnant SCCY has sexual encounters will multiple partners. Expectant SCCY are therefore discouraged from having multiple sexual partners.

Female FGD, 14–17 years: *If you sleep with other men while pregnant you might lose the baby because the bloods are different*.

Male FGD, 18–24 years: *Another thing that leads to abortion is mixing men*, *when you are pregnant and then another man comes (sex) and spoils it (pregnancy)*. *The blood starts to spill (miscarriage)*.

Even though it commonly occurs, abortion is not an acceptable practice among SCCY, and is illegal in Kenya. A majority of abortion cases are reported to occur among SCCY girls who are under the influence of drugs (glue). The strong stand against abortion is influenced by the street community’s religious beliefs that abortion is sin. Those who are discovered to have committed the act are severely punished by street member or referred to law enforcers for possible incarceration. SCCY however believe that incarceration is not an effective approach to addressing the problem. This is because female SCCY are normally released from jail and end up back on the streets where they continue in the cycle of unwanted pregnancies and abortion. Hence, physical abuse by street members is deemed as the best form of punishment.

Male FGD, 18–24 years: *You know*, *I had married a girl from the streets*. *She got pregnant by me*. *She then terminated the first*, *second*, *and third pregnancy*. *Like the bible says ‘abortion is sin’*. *One day I watched as she came to the base… I looked at her and I cried*. *We were still living together*. *Then another feeling came inside me (referring to anger) and I broke her leg because she had aborted four times and I didn’t know what to do*. *I thought to break her neck but then I thought NO*…

Female FGD, 14–17 years: S*he is beaten (referring to a female SCCY who has performed an abortion)*. *She does that after sniffing glue …*. *if she is arrested she will still be released when she sniffs glue she acts like a mad person*

Male FGD, 18–24 years: *You take her to jail or beat her… We beat her because when we take her there (referring to jail)*, *they will just release her and she comes back and continues to abort and abort*.

### Unwanted Infants

Since there are heavy penalties imposed on female SCCY who abort, the majority prefer to carry their pregnancies to term and deal with the infant once they are born.

#### Care centers

Care centers including orphanages are viewed as a safe haven for unwanted children. It also eliminates the sense of guilt that some female SCCY may experience if they terminated their pregnancies. In addition, SCCY reported that they appreciated the arrangement established with these care centers because it provided them with the opportunity to see and interact with their children on a regular basis.

#### Selling of infants

Therefore some female SCCY opt to sell their unwanted babies to willing individuals. It was not clear who these individuals are; however, the hospital setting was reported as one of the places where willing buyers are easily identified.

Female FGD, 14–17 years: *They sell them…if you go to the hospital you will find those who buy babies*. *They ask you how much you want; the nurse can tell you that you have a beautiful baby and tell you she has a certain amount of money*.

Male In-depth Interview, 21 years: *They even give birth but the problem is now taking care of the child*. *You will find that they give birth and give out the child to another girl to go with the child on the streets*. *So the other girl who goes with the child will give the child her breast and this makes the child suffer*. *They also keep on hitting the children*. *So in most cases they give the children to anybody because they cannot take care of the children*.

#### Abandon infants

Female SCCY also abandoned their unwanted infants on the street. The desire to start a new sexual relationship coupled with drug and alcohol abuse are some of the factors associated with this act.

Female In-depth Interview, 21 years: *Yes*. *And some will go and drink alcohol so that they can pretend that they are drunk then they leave the child pretending they forgot or you go at Lengut where we used to sleep*, *leave the child there and tell the watchman to help you look after the child so that you can go and look for money but when you go you never come back*.

Male In-depth Interview, 14 years: *They can give birth but throw the baby in the dustbin or leave the baby by the roadside and someone else picks up the baby and raises it*.

#### Infanticide

Female SCCY sometime murder their infants through acts that reflect their psychological and mental health status.

Male FGD, 11–13 years: *or you give birth then kill the baby…mmmh*…*she throws the baby inside a toilet…aha…buries the baby alive…*

Male In-depth Interview, 22 years: *They don’t abort*. *They just give birth and kill because if you abort you cannot stay at the base*. *You will be beaten*. *So they cannot abort maybe they just kill and that doesn’t mean that they just kill openly*. *You do it in a secret where you can go with the child somewhere you kill and come pretending that you are crying when you just know what you have done*.

Female In-depth Interview, 17 years: *There are others who kill*. *They just give birth to the child very well*. *You can imagine with all the labour pain then they get hold of the child’s neck and they strangle the child*. *So you even wonder what comes in their mind when they do such a thing*! *I would rather they even just throw the child instead of strangling*.

### Preventing pregnancies

A few health institutions waive fees for health care services provided to SCCY including reproductive care making family planning services easily available to them. SCCY were therefore familiar with some of the contraceptives available but described them by the mode of application such as pills, injection, and condoms. A majority of the male SCCY did not approve of their female SCCY using these methods because they expected their girls or wives to bear them children. They had a number of perceptions associated with the use of these contraceptives. Fear of infertility deters females SCCY from opting for injectable contraceptives. In addition, condoms are viewed as not pleasurable with the majority of male SCCY disliking them. Some male SCCY also fear that the fluids in condoms may affect their sexual organs and lower their fertility. They therefore prefer using polythene bags to condoms.

Female In-depth Interview, Barracks Leader, 20 years: *There are some who do not want to get pregnant and go for an injection…some street girls are jealous*, *so they will go and tell her husband that his wife was injected and for a certain period she cannot give birth*. *That shows that they want your husband so the husband will beat the wife seriously and even want to kill her*. *It will now force the girl to go back to the hospital for that to be reversed*.

Female In-depth Interview, 15 years: *Some girls also say that they cannot use family planning methods because they say that the injection makes people lose their fertility*. *And some also say that if they use a condom*, *it makes sex not sweet…The girls prefer condoms but boys don’t want*. *They (referring to the SCCY boys) have now ended up impregnating all of my friends*. *There are only two who are not pregnant*.

Male In-depth Interview, Barracks Leader, 24 years: *Because they tell us (referring to health educators) that condoms should not be in contact with oil but it already has oil in it*. *As for me*, *I wouldn’t advise others to do as I do*, *but what I know is that the hole (referring to vagina) that I enter because it’s not like I just get a woman and I enter (sex)*. *When you find a woman and she tells you to buy a condom*, *you buy then you go to the base (SCCY meeting place)*, *and because we are known there*, *we take two polythene bags*, *we put them on before we put on the condom*. *They say you leave some space at the front of the condom*, *and then imagine you did leave some sweat at your front*, *so the oil gets into your ‘deki’ (penis)… So you find that*, *with the condom only even if you wanted to go 5 rounds you will only go once because of the oil… You know when you pump*, *your blood also gets hot and thus the ‘deki’ (penis) becomes hot too and that’s when you get the oil…*

There are other methods SCCY use to prevent pregnancy during their sexual encounters which included unprotected anal sex and sex with small boys. Even though the use of these approaches would not result in pregnancy, engaging in these sexual acts has implications for the health outcomes of this vulnerable group.

Female In-depth Interview, 15 years: *They don’t take care of themselves*. *Some of them even assume that the boys they are doing sex with are very young and therefore they cannot get pregnant*.

Male 24 years: *But the thing that also makes women not get pregnant is because they engage in anal sex*.

## Discussion

Despite the harsh street environment, SCCY have a strong desire to identify with the larger society by going through conventional life experiences such as marriage and child bearing. These findings are consistent with other studies conducted in developed countries.[36,37] Evident were the gender differences around the desire to have children. Male SCCY were more focused on their social status in the street community, while for females, having children was more of a street coping mechanism. Like other adolescent girls, SCCY girls bear a myriad of health risks in the continuum of pregnancy.[30] More significantly, female SCCY enter pregnancy with a myriad of other health challenges including nutritional deficiencies, drug abuse, sexual transmitted diseases, and mental health issues [9,11,12,17,32,38,39] that have grave implications for their pregnancy outcomes.[26,30] Furthermore, children born of these adolescent mothers face even greater health risks following these maternal and environmental factors. Our findings bring to light the desire for street youth to have families and this could be used to develop appropriate interventions for this vulnerable group.

While some pregnancies are desired and planned, a number of them are unwanted[30]. These are pregnancies that result from rape, transactional sexual encounters with unfamiliar male partners or following partner conflict (SCCY) during their pregnancy term. In the event of lack of partner support, abortion is a common option. Compared to adults in some developing countries, adolescents are less likely to obtain legal and safe abortions.[30] More specifically in Kenya, there are no clear guidelines about legal abortion [40] which promotes unsafe abortion practices among this vulnerable group.[28,40]

Surprisingly, the street community prohibits abortion; a belief that stems from a strong religious background held by the majority. Women who abort are physically abused by their peers since they believe that current law enforcing structures are ineffective. Despite this, unsafe abortion either assisted or self-induced, occurs and includes unorthodox practices such as ingesting of harsh substances, pricking or kicking of the fetus, and seeking services from unlicensed health workers and medicine men. Interestingly, female SCCY are willing and can afford to pay up to Ksh 5,000 (= $56 USD) to have an assisted abortion; money which may have been obtained through transactional sex. Unlike the perceptions of older women in Kenya,[41] SCCY believe that abortion is safer when induced early in the pregnancy (first trimester) and hence are more likely to induce abortion during this period. In addition, having multiple sexual partners during pregnancy is viewed as a form of abortion. Unfortunately, this perception also increases the risk of sexually transmitted infections including HIV. The lack of social, psychological, and financial support drive female SCCY to take drastic measures to terminate their pregnancy. Establishing practical maternal health interventions for this population that incorporates safe abortion options may be critical to reducing the risk associated with unsafe abortion practices that they adopt.

Gender inequalities around reproductive health issues were evident. Male SCCY expressed dominance over their female counterparts. In this context, the desire for children is mainly driven by male SCCY who restrict female SCCY from using contraceptives and who have a preference for male children. Even though abortion is prohibited, female SCCY desiring to establish new sexual relationships with male SCCY, are expected to get rid of their pregnancies and/or children from previous relationships. Lack of agency among female SCCY promotes unhealthy reproductive choices and health interventions will need to identify ways of empowering female SCCY to make informed decisions about their reproductive health.

While some female SCCY may opt to carry their pregnancies to term to avoid street penalties associated with abortion, infants born of SCCY are sometimes sold, abducted, abandoned, or murdered by their mothers. Child trafficking is a serious offense under Kenya’s Counter-Trafficking in Persons Act of 2010, yet there lacks a formal mechanism for identifying victims of trafficking among vulnerable populations.[42] Child protection services need to be augmented and trafficking laws enforced to protect those most vulnerable. In addition, we believe that a combination of drug abuse and psychological and mental health issues common among SCCY mothers[11,12,38] may significantly contribute to the acts of infant murders reported. Health programs will need to critically evaluate the dynamics created by the lack of adolescent reproductive healthcare services and the unfriendly street environment, in the effort to develop tailored interventions for SCCY. Promoting drug rehabilitation programs that address mental health issues among this group of mothers is also fundamental.

While care centers including orphanages are available to take care of abandoned and vulnerable SCCY infants, they may be contributing to the cycle of unwanted pregnancies among this population. This is because SCCY are assured that the care centers will easily accept their babies while enhancing parent-child interaction. Furthermore, there appears to be a lucrative market for unwanted SCCY infants, with reports of healthcare workers abducting SCCY babies during hospital deliveries. We believe that there may be a general assumption that all street pregnancies are unwanted, hence individuals take advantage of the vulnerability of female SCCY. There is a need to develop appropriate support programs for SCCY mothers who may be willing to take care of their children but lack the necessary support or ability to do so. This should be integrated with interventions that empower SCCY girls to make informed decisions in order to avoid the cycle of unwanted pregnancies. In addition, law enforcers should address the black market that promotes the illegal selling of babies.

It was encouraging to note that the street community has access to free reproductive healthcare services. SCCY were aware about some of the contraceptive methods available. However, the adoption of these methods was heavily influenced by the male SCCY and confounded by the desire to have children and misconception around injectable contraceptives and condom use. To avoid pregnancy, female SCCY opted for other approaches such as unprotected anal sex and intercourse with small boys. Unfortunately, this continues to promote early sexual debut and heightens the risk of sexual transmitted diseases. There is a need for more reproductive health awareness among this community to address the misconceptions that exist with use of contraceptives. In addition, youth friendly reproductive services that take into account the unique social environment of SCCY should be encouraged.

Finally, efforts to repatriate SCCY are needed. However in doing so, it is critical to consider the reasons why these SCCY left home in the first place. In some cases, repatriation may not be appropriate due to the trauma and violence SCCY faced in the hands of their parent/guardians[10]. Hence programs that focus on providing SCCY with healthy home environments including foster homes for this vulnerable group are needed.

This study is not without limitations. We were unable to conduct FGDs with females aged 11–13 years. This was due to the low numbers of this group living on the streets that made it difficult to recruit them into the study. In addition, the sampling procedure limits the generalizability of the findings. Moreover, youth who agreed to participate may have been systematically different from those who did not participate in the study or those whom we could not recruit (girls 11–13 years). Social desirability bias could have also influenced the way SCCY responded to sensitive questions regarding sexual practices. Despite these limitations, this is the first study in a resource-constrained setting that provides insight into the perceptions held by SCCY about pregnancy. These findings have implications for interventions and policies that aim to promote the overall wellbeing of SCCY in Kenya and particularly those related to reproductive health and family planning. Further studies are need to provide more insight into the sexual and reproductive health issues among this population.

In conclusion, our study highlights that SCCY perceived more advantages than disadvantages to getting pregnant. Unfortunately, their perceptions put significant pressure on female SCCY to become mothers when they are not physically, psychosocially or financially prepared. With the lack of support, SCCY girls opt for unorthodox approaches to terminate their pregnancy or get rid of their unwanted babies. Although easily available, contraception is not encouraged and associated with a number of misconceptions. Public health programs will need to develop youth friendly interventions that provide tailored maternal health services including safe abortion options for SCCY girls. More health education and life skills programs are also needed to promote safe sex practices and informed decisions among this vulnerable group.
